# Vasoconstrictor and Pressor Effects of Des-Aspartate-Angiotensin I in Rat

**DOI:** 10.3390/biomedicines10061230

**Published:** 2022-05-25

**Authors:** Rosemary Wangensteen, Manuel Gómez-Guzmán, Inmaculada Banegas, Isabel Rodríguez-Gómez, Rosario Jiménez, Juan Duarte, Joaquín García-Estañ, Félix Vargas

**Affiliations:** 1 Area of Physiology, Department of Health Sciences, University of Jaén, 23071 Jaén, Spain; rwangens@ujaen.es (R.W.); ibanegas@ujaen.es (I.B.); 2 Department of Pharmacology, University of Granada, 18071 Granada, Spain; mgguzman@ugr.es (M.G.-G.); rjmoleon@ugr.es (R.J.); jmduarte@ugr.es (J.D.); 3 Department of Physiology, University of Granada, 18071 Granada, Spain; isabelrg@ugr.es; 4 Department of Physiology, Faculty of Medicine, IMIB, University of Murcia, 30120 Murcia, Spain; jgestan@um.es

**Keywords:** renin-angiotensin system, des-aspartate-angiotensin I, vascular reactivity, kidney, hypertension

## Abstract

This study investigated the vasoactive effects of des-aspartate-angiotensin-I (DAA-I) in male Wistar rats on whole body vascular bed, isolated perfused kidneys, and aortic rings. Dose–response curves to DAA-I were compared with those to angiotensin II (Ang II). The Ang II-type-1 (AT1) receptor blocker, losartan, was used to evaluate the role of AT1 receptors in the responses to DAA-I. Studies were also conducted of the responsiveness in aortic rings after endothelium removal, nitric oxide synthase inhibition, or AT2 receptor blockade. DAA-I induced a dose-related systemic pressor response that was shifted to the right compared with Ang II. Losartan markedly attenuated the responsiveness to DAA-I. DAA-I showed a similar pattern in renal vasculature and aortic rings. In aortic rings, removal of endothelium and nitric oxide inhibition increased the sensitivity and maximal response to DAA-I and Ang II. AT2 receptor blockade did not significantly affect the responsiveness to DAA-I. According to these findings, DAA-I increases the systemic blood pressure and vascular tone in conductance and resistance vessels via AT1 receptor activation. This vasoconstrictor effect of DAA-I participates in the homeostatic control of arterial pressure, which can also contribute to the pathogenesis of hypertension. DAA-I may therefore be a potential therapeutic target in cardiovascular disease.

## 1. Introduction

**Figure 1:**
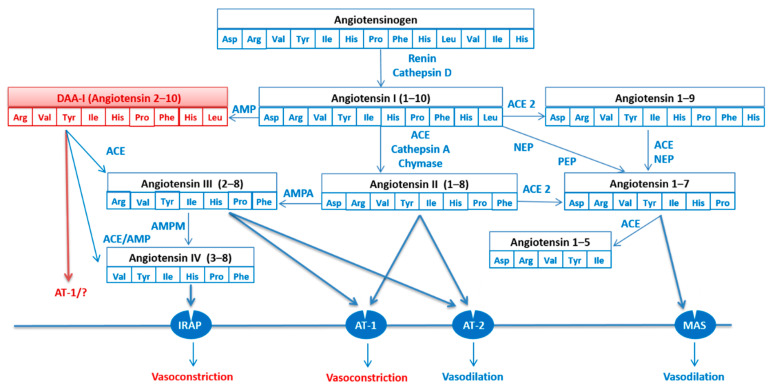
The renin–angiotensin system (RAS). Renin increases angiotensin I (Ang I) formation from angiotensinogen and ACE/chymase induces angiotensin II (Ang II) formation from Ang I. Ang I is converted to angiotensin (1–7) and angiotensin (2–10) or DAA-1. Angiotensin II and Angiotensin III stimulate AT-1 and AT-2 receptors. Angiotensin 1–7 bind to the MAS receptor. The ACE2-Ang-(1–7)-Mas axis is now considered a putative counter-regulatory system to the ACE-AngII-AT1R axis. Angiotensin IV stimulates AT-4 receptors or insulin-regulated aminopeptidase (IRAP). Effects mediated via AT-2 and MAS receptors generally oppose those of AT-1. Abbreviations: ACE: angiotensin converting enzyme. AMP: aminopeptidase. AT-1: angiotensin II type 1 receptor. AT-2: angiotensin II type 2 receptor. DAA-A: Des-aspartate-angiotensin-I. IRAP: insulin-regulated aminopeptidase. NEP: neutral endopeptidase. PEP: prolyl endopeptidase. “?” means that it is possible that there is other mechanisms other than AT-1.

The renin–angiotensin system (RAS) plays a central role in cardiovascular pathophysiology. RAS is considered an endocrine system in which angiotensin II (Ang II), the main effector peptide, is produced by the sequential enzymatic cleavage of angiotensinogen. Systemic Ang II increases arterial blood pressure (BP) through Ang II type 1 (AT1) receptor-dependent arterial vasoconstriction and reductions in renal salt and water excretion via extrarenal and intrarenal mechanisms [1,2]. Changes in the RAS are implicated in the pathophysiology of many cardiovascular diseases. Ang II is cleaved by aminopeptidases A and N to Ang III and Ang IV, respectively. This is also the case for other Ang fragments, including the heptapeptide Ang 1–7, derived from Ang I by tissue endopeptidases [3], and the octapeptide Ang A, generated from Ang II by enzymatic decarboxylation of Asp1 [4,5]. These metabolites have long been considered of little importance but have displayed biological activity (Figure 1) [5,6,7,8].

Ang I is mainly metabolized to Ang II by the enzymatic action of angiotensin-converting enzyme (ACE), although it is also metabolized to des-aspartate-angiotensin-I (DAA-I or Ang 2–10) by aspartate or glutamate aminopeptidase activity (APA). Forty years ago, García del Río et al. [9] identified DAA-I in rat blood and confirmed that it is a substrate for ACE, suggesting that at least part of the Ang III in rat blood may be formed by the action of ACE on endogenous DAA-I. In this way, DAA-I is generated from Ang I through the enzymatic decarboxylation of Asp1 by APA, and the removal of His-Leu by ACE yields Ang III (Figure 1).

In a rat study, Campbell et al. [10] reported that the predominant angiotensin peptide in plasma was Ang I, with Ang II levels being around half of Ang I levels. DAA-I concentrations were similar to Ang II levels, and Ang III levels were around half of Ang II levels. In a later investigation, Sim and Qui [11] found similar plasma concentrations of DAA-I, Ang I, Ang II, and Ang III in both Sprague–Dawley and Wistar-Kyoto (WKY) rats. DAA-I has been demonstrated to be active when administered orally [12,13,14,15], showing a rapid systemic metabolism when infused into the circulation or orally administered in humans [14,16].

Although DAA-I has been found to contract rat aortic rings [17], it has been reported to exert greater action against the vasoconstrictor effects of Ang II and Ang III in the brain and peripheral circulation. In this way, DAA-I attenuated the action of Ang III in the aortic rings of rabbits [18] and in the renal and mesenteric vasculature of spontaneously hypertensive rats (SHRs) and WKY rats [19]. DAA-I has been shown to inhibit the deleterious actions of Ang II and its intracerebroventricular administration also attenuated the central pressor actions of Ang II and III in SHR and WKY rats [20,21]. It was later found that DAA-I reduces the pressor action of Ang II in the renal vasculature of SHR and WKY rats [22]. In addition, DAA-I has demonstrated antioxidant and anti-inflammatory actions in gamma-irradiated mice [13].

As reported above, DAA-I is present in human plasma at concentrations that may have relevant effects, possibly counteracting the effects of Ang II and Ang III. However, the direct effects of this peptide are not well characterized. With this background, the objectives of this study were to investigate the direct vasoactive effects of DAA-I on the complete systemic vascular tree, the renal vasculature in the isolated perfused kidney (resistance vessels), and aortic rings (conductance vessels). Further objectives were to determine the role of AT1-receptors in the responses to DAA-I and evaluate the modulatory role of the endothelium-NO pathway.

## 2. Materials and Methods

### 2.1. Animals

Forty-eight male Wistar rats (18–20 weeks old) born and raised in the experimental animal service of the University of Granada were randomly assigned to one of the experimental groups (*n* = 6 in each group). Experiments were conducted in accordance with European Union guidelines on the ethical care and use of laboratory animals and were approved by the Animal Experimentation Ethics Committee of the University of Granada (permit no: 03-CEEA-OH-2013).

### 2.2. Experimental Protocols

Experiments were designed to examine the effects of DAA-I on the whole body, isolated perfused kidneys, and aortic rings of male rats. Dose–response curves to DAA-I were compared with the responsiveness to Ang II administered at the same doses in the three preparations. The responsiveness to DAA-I and Ang II was also studied in the presence of the AT-1 receptor blocker losartan in order to analyze the contribution of the AT-1 receptor to the pressor or vascular effects of these agents.

#### 2.2.1. Experiment 1. Whole Body Response to DAA-I and Ang II

After anesthetizing the rats with 2.0 mL/kg i.p. thiobutabarbital (Inactin), a catheter was introduced into the right carotid artery for direct BP measurements and into the right jugular vein for drug administrations. Then, the arterial catheter was connected to a transducer (MacLab, AD Instruments, Hastings, UK) for continuous 60 min BP recording in the anesthetized rats. The mean BP during the last 20 min was considered the baseline value for intergroup comparisons. A dose–response curve to i.v. bolus of DAA-I (0.01–2 µg/kg) was obtained in the whole rat vascular bed and compared with the curve obtained for Ang II (0.01–2 µg/kg). Both dose–response curves were also constructed in the presence of an i.v. bolus of losartan (10 mg/kg) in the different preparations (*n* = 6 each group). Losartan or saline was i.v. injected at 30 min before obtaining the dose–response curves. Agonists were given in increasing doses at intervals of 15 min. Each dose–response curve was performed in a different animal; therefore, 24 animals were used in this experiment.

#### 2.2.2. Experiment 2. Renal Response to DAA-I and Ang II in the Isolated Rat Kidney

Animals were anesthetized with pentobarbital sodium (40 mg/kg, intraperitoneal, i.p.). The kidney was removed and perfused at a constant flow rate (5 mL/g kidney weight/min) with Tyrode’s solution at 37 °C, as previously reported [23]. The kidney was placed in a chamber (kept at 37 °C) that collected perfusate lost from the renal vein, which was not recirculated and was removed. Rats were then killed with an overdose of pentobarbital sodium. Renal responses were recorded as changes in renal perfusion pressure (RPP) measured with a pressure transducer (MacLab, AD Instruments, Hastings, UK) connected to the perfusion circuit downstream from the perfusion pump. Dose–response curves were made to DAA-I and Ang II, in this order, from 10^−9^ to10^−6^ M for both agonists. They were performed under baseline conditions (*n* = 6) and, in separate preparations (*n* = 6), after the addition of 10^−6^ M of losartan to the perfusate at the start of perfusion. A total of 12 animals were used for these experiments.

#### 2.2.3. Experiment 3. Response to DAA-I and Ang II in the Presence and Absence of Endothelium in Aortic Rings

Rats were anesthetized and sacrificed by cervical dislocation. Rings were dissected from the descending thoracic aorta, mounted in organ chambers filled with Krebs solution (components in mM: glucose, 11; NaHCO_3_, 25; NaCl, 118; KCl, 4.75; CaCl_2_, 2; KH_2_PO_4_, 1.2; MgSO_4_, 1.2), and aerated at 37 °C with carbogen (95% O_2_ and 5% CO_2_). Each ring was stretched to a resting tension of 2 g using two L-shaped stainless-steel wires inserted into the lumen and connected to the chamber and an isometric force-displacement transducer (Letigraph 2000, Letica S.A., Barcelona, Spain), and it was then equilibrated for 60–90 min, as previously reported by our group [24]. In some rings, the endothelium was mechanically removed by rubbing the intimal surface of the ring with a metal rod. The absence of endothelial cells was confirmed by the lack of acetylcholine relaxation (10^−6^ M) in aortic rings previously contracted with phenylephrine (10^−7^ M).

After equilibration, concentration–response curves to DAA-I (10^−9^ M to 10^−5^ M) and Ang II (10^−9^ to 10^−5^ M) were constructed for individual rings by adding cumulative concentrations of each drug in the presence (*n* = 6) and absence (*n* = 6) of losartan (10^−6^ M), the AT2 receptor antagonist PD123319 (10^−6^ M), or the nitric oxide synthase (NOS) inhibitor N^ω^-nitro-*L*-arginine methyl ester, L-NAME (10^−4^ M) to evaluate the potential modulatory role of AT2 receptor and of nitric oxide in the vascular effects. Data were expressed as % of the contraction produced by 80 mM KCl.

### 2.3. Drugs

The following drugs were used: pentobarbital purchased from Serva (Heidelberg, Germany); inactin, losartan, and L-NAME from Sigma-Aldrich (St. Louis, MO, USA); DAA-I and Ang II from Bachem (Bubendorf, Switzerland); and PD123319 (Tocris, Minneapolis, MN, USA).

### 2.4. Statistical Analyses

A nested-design analysis of groups and doses was used to compare dose–response curves; the design had two fixed effect factors (group and dose) and one random effect factor (whole rat, kidney, or aortic ring), with this factor being nested in the group. Data were compared using a factorial ANOVA for repeated measures, taking each rat as subject, and group and dose as between-subjects factors. Interactions between factors were analyzed using the Bonferroni method. *p* values < 0.05 were considered statistically significant. SPSS v 20.0 (IBM, Chicago, IL, USA) was used for data analyses.

## 3. Results

### 3.1. Whole Body Response

**Figure 2:**
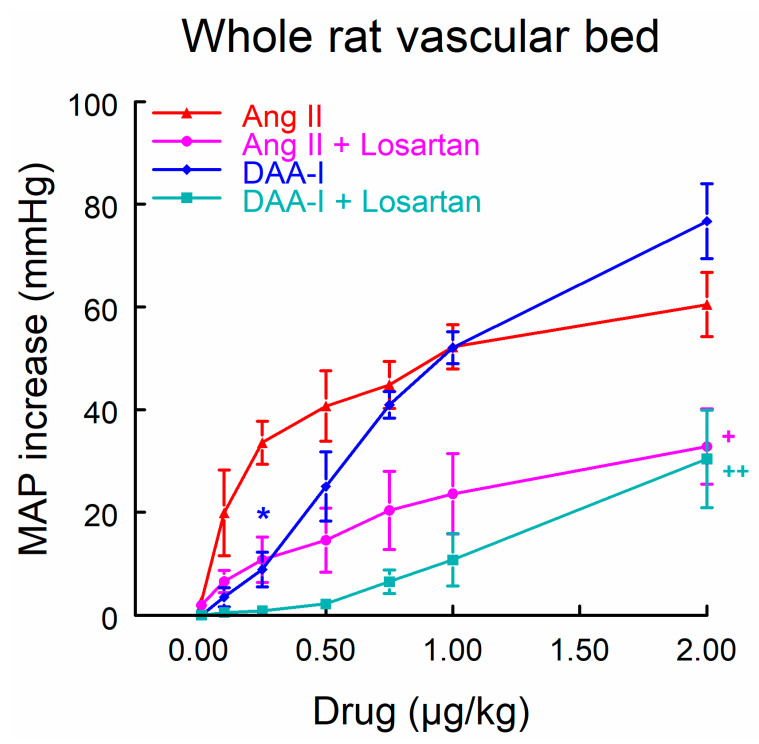
Dose–response curves to DAA-I and to Ang II with or without losartan in whole rat vascular bed. Data were compared using a factorial ANOVA for repeated measures, taking each rat as subject, and group and dose as between-subjects factors. Interactions between factors were analyzed using the Bonferroni method. * *p* < 0.01 versus the same dose of Ang II. + *p* < 0.05, ++ *p* < 0.01 along the curve versus its respective dose–response curve without losartan. Data are means ± standard error of the mean (SEM). *n* = 6 each group. MAP = mean arterial pressure.

Figure 2 shows that the intravenous administration of DAA-I produced a dose-related systemic pressor response. The dose–response curve to DAA-I was characterized by a shift to the right in comparison to the curve obtained with Ang II, showing a lower response to low or medium doses and a similar response to high doses. Intravenous (i.v.) administration of losartan produced no difference in baseline BP but markedly attenuated the responsiveness to DAA-I and Ang II, whose dose–response curves maintained the same differences and similarities between them.

### 3.2. Vasoconstrictor Response in the Renal Vasculature

**Figure 3:**
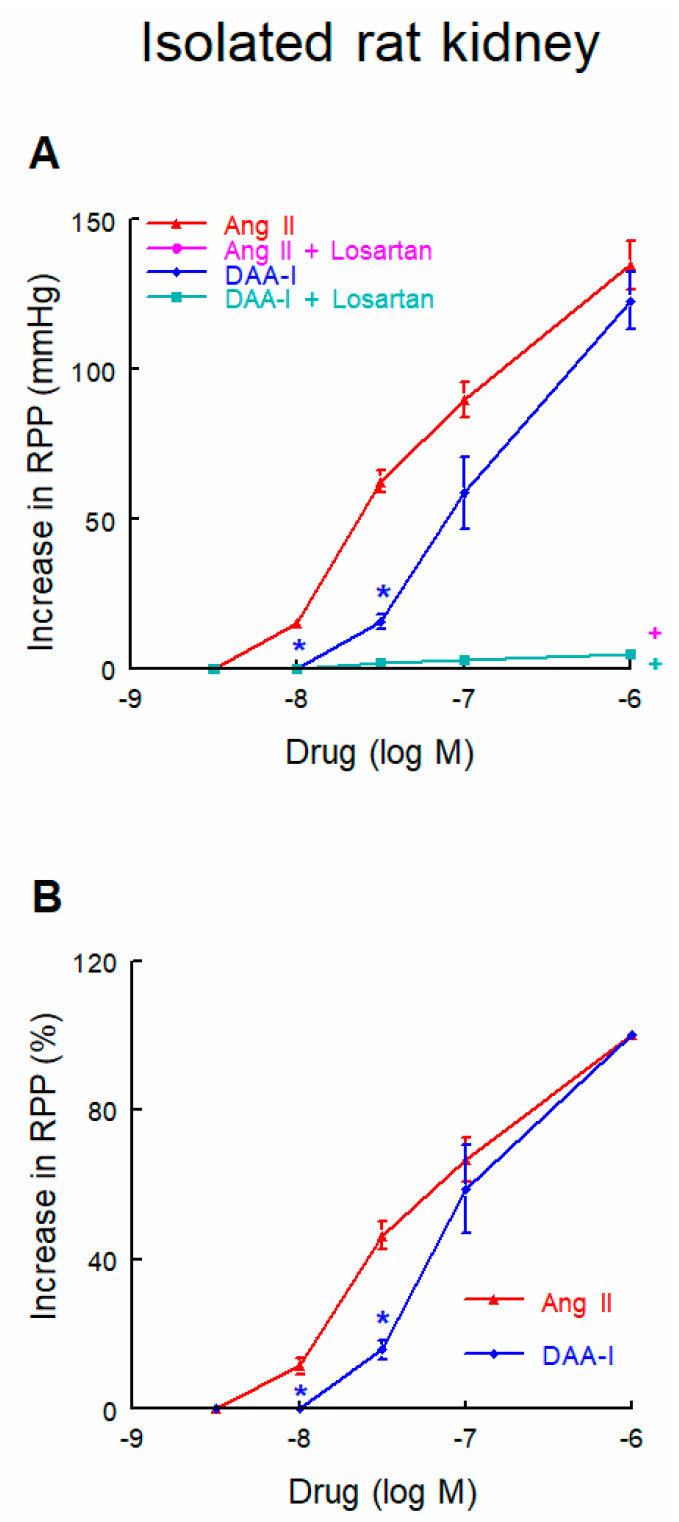
Dose-response curves (**A**) and percentage of maximal responses (**B**) to DAA-I and to Ang II in isolated perfused rat kidney. Data were compared using a factorial ANOVA for repeated measures, taking each rat as subject, and group and dose as between-subjects factors. Interactions between factors were analyzed using the Bonferroni method. * *p* < 0.01 versus the same doses of Ang II. + *p* < 0.001 along the curve versus its respective dose-response curve without losartan. Data are means ± SEM. *n* = 6 each group. RPP = renal perfusion pressure. Ang II + Losartan trace is not visible because responses were similar to DAA-I + Losartan.

DAA-I administration produced a vasoconstrictor renal response (Figure 3). The dose–response curve to DAA-I was characterized by a shift to the right, while comparison with the dose–response curve to Ang II showed a lower response to low or medium doses and a similar maximal response. Administration of losartan in the perfusate markedly suppressed the pressor response to both DAA-I and Ang II.

### 3.3. Effect on Aortic Ring Tone

**Figure 4:**
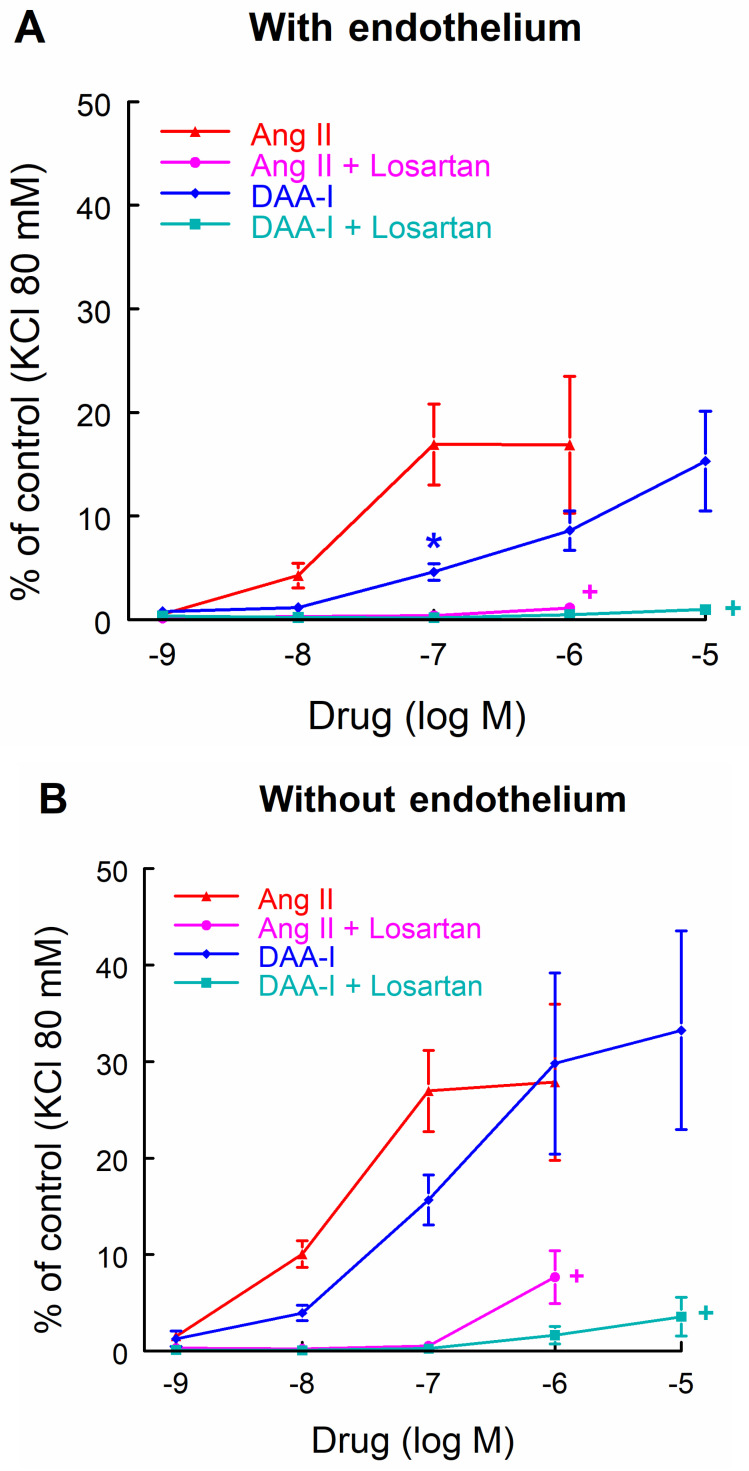

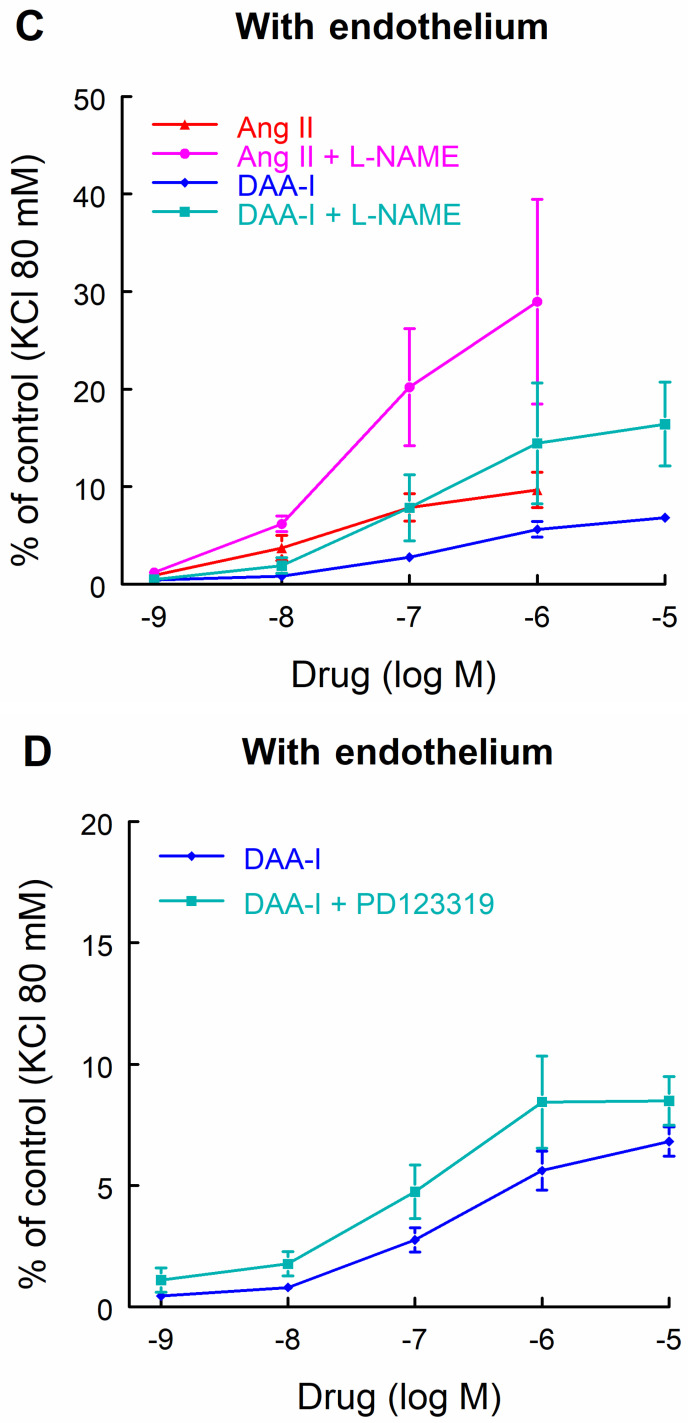
Concentration-response curves to DAA-I and to Ang II in aortic rings with (**A**,**C**,**D**) or without (**B**) endothelium in the presence of losartan (**A**,**B**), L-NAME (**C**), or PD123319 (**D**). Data were compared using a factorial ANOVA for repeated measures, taking each rat as the subject, and group and dose as the between-subjects factors. Interactions between factors were analyzed using Bonferroni method. * *p* < 0.01 versus the same dose of Ang II. + *p* < 0.001 along the curve versus its respective dose-response curve without losartan. Data are means ± SEM. *n* = 6 each group. KCl = potassium chloride.

Administration of DAA-I augmented the vascular tone in aortic rings (Figure 4). The concentration–response curve to DAA-I and its comparison with the curve to Ang II were similar to the above observations in the intact rat and renal vasculature. Pharmacological inhibition of AT1 with losartan suppressed the vasoconstrictor response of DAA-I and Ang II (Figure 4A). Removal of the endothelium increased the sensitivity and maximal response to DAA-I and Ang II, suppressing the differences between these drugs at low and medium concentrations (Figure 4B). Similar effects were observed when aortic rings with endothelium were incubated for 20 min with the NOS inhibitor L-NAME (Figure 4C). The presence of PD123319, an AT2-receptor blocker, in the chamber did not significantly change the responsiveness to DAA-I (Figure 4D), indicating that the observed pressor effect of DAA-I is not AT2 receptor-mediated. Taken together, these results show that DAA-I has very similar effects to those evoked by Ang II, and that differences between them are diminished when the endothelium is absent or NOS is inhibited.

## 4. Discussion

To our best knowledge, this is the first report to demonstrate that DAA-I acts as a potent vasoconstrictor in three preparations that represent the main part of the cardiovascular system involved in BP control and, therefore, in the pathogenesis of hypertension. These include the complete systemic vascular tree and the conductance and resistance vessels of the renal vasculature. Resistance vessels were selected because of the major role of renal hemodynamics in blood pressure control and in renal sodium handling [25,26]. We also demonstrate that the vasoconstrictor and pressor responses produced by DAA-I are mediated via the AT_1_ receptor, with scant involvement of the AT_2_ receptor. Responses to DAA-I were lower than responses to Ang II at low and intermediate doses, but the two agents induced similar responses at maximal doses in in vivo and in vitro preparations. Taken together with previous observations of similar plasma DAA-I and Ang II levels in normotensive rats [10], these findings indicate a physiological role for DAA-I, especially at high levels, in the promotion of vasoconstrictor effects.

In contrast, the decapeptide Ang I only appeared to have significant biological activity in the central nervous system and pulmonary vascular bed, where it produced dose-dependent increases in lobar arterial pressure under ACE inhibition [27]. However, Ang I was found to have little or no activity in smooth muscle isolated from other vascular beds [28,29].

RAS is now considered to have two opposite arms. The pressor arm is primarily formed by Ang II, which activates the Ang II type 1 (AT1) receptor (AT1R), with vasoconstrictive, proliferative, hypertensive, oxidative, and pro-inflammatory effects [30]. The depressor arm mainly comprises AT2 receptors Ang-(1–7) and activates the Mas receptor, opposite to the effects mediated by AT1R activation [31]. These findings indicate that DAA-I should be included as a new component in the group of RAS agonists that activate the AT1 receptor, alongside Ang II, Ang-(1–12), Ang III, and Ang IV [32,33].

Various authors have proposed that the main action of DAA-I is the counteraction of Ang II-mediated vasoconstriction [12,34,35]. For instance, Sim [12] describes DAA-I as a physiological antagonist to Ang II that is capable of binding to angiotensin AT1 receptors, releasing prostaglandins [12,36]. In this way, DAA-I antagonized the actions of Ang II [20,34] and attenuated Ang II pressor action in isolated perfused kidney and mesenteric vascular bed of both SHR and WKY rats [22] as well as reducing the pressor action of Ang III [19] via AT1 receptors [37]. Moreover, DAA-I decreased Ang II-induced contractions in the aorta of SHRs [35]. Given that the aforementioned authors [12,20,22,35] had performed a dose–response curve to Ang II before obtaining the curve to DAA-I, these effects may result from the desensitization of AT1 receptors, as observed when successive dose–response curves to Ang II are performed.

In fact, data obtained from electrically contracted endothelium-denuded rabbit pulmonary arteries showed that DAA-I acted as an agonist of the AT_1_ receptor [38].

Plasma concentrations of Ang III and Ang II were found to be the same in conscious dogs [39] and Ang III is virtually equipotent with Ang II in eliciting vasoconstriction in the kidney and in releasing aldosterone from the adrenal cortex [29,40]. The kidneys of SHRs displayed a higher activity of aminopeptidase A, the main enzyme that generates DAA-I and hydrolyzes Ang II to Ang III [41], suggesting that DAA-I and Ang III may contribute to the pathogenesis of genetic hypertension.

Kono et al. [16] observed a striking rise in BP and an increase in plasma aldosterone concentrations in healthy men after an intravenous infusion of DAA-I, which were abolished by ACE blockade. Similar effects were observed in rats, in which pressor and steroidogenic actions of DAA-I were dependent on the hydrolysis of (DAA-1) to angiotensin III [42]. Uninephrectomized fawn-hooded hypertensive rats treated with low and high doses of DAA-I also showed a rise in BP [43], whereas Lee et al. [14,15] observed no BP changes in healthy subjects given a single oral dose of DAA-I at different concentrations. Therefore, besides the direct effects of DAA-I reported in the present study, it can also be considered a precursor for the biological effects of angiotensin III.

Alternative pathways of conversion of Ang I to DAA-I may maintain the vasoconstrictor tone when Ang II production is blocked by the administration of ACE inhibitors. Consistently, Campbell et al. [10] reported that perindopril increased DAA-I levels 13-fold in plasma of normotensive rats, and Sim and Qui [11] showed that treatment with ACE inhibitors significantly increased plasma DAA-I levels in SHRs and hypertensive patients. Hence, the present data provide experimental support for the recent therapeutic strategy to produce a double blockade of the RAS by ACE inhibition [44,45]. This produces an increase in kinins [46], exerting vasodilator and natriuretic effects in combination with AT_1_ blockade, which can interact with DAA-I activity when Ang II production is reduced.

The present aortic ring findings demonstrate that the absence of endothelium increases the sensitivity of the vasoconstrictor response to DAA-I and that the administration of the AT2 blocker potentiates, although without reaching statistical significance, the vasoconstrictor response to DAA-I. This phenomenon may be relevant in the vast majority of cardiovascular diseases that course with endothelial dysfunction, in which DAA-I may play an important pathogenic role. DAA-I may therefore be a potential therapeutic target in cardiovascular diseases. Finally, this study lacks information on many other activities developed by RAS components via AT1 receptor activation, including proliferative, oxidative, pro-inflammatory, and profibrotic effects [30].

## 5. Conclusions

In conclusion, the present data indicate that DAA-I is a full agonist of AT1 receptors. These findings, together with observations of similar plasma DAA-I and Ang II levels in normotensive rats, contribute evidence that DAA-I has a physiological role in cardiovascular function and a pathogenic role in cardiovascular disease. It may be particularly relevant in patients treated with ACE inhibitors, which increase plasma levels of DAA-I. Further research is warranted on the possible role of DAA-I in the impact of angiotensins on the broad central nervous system and in their systemic effects, especially in relation to aldosterone secretion and the regulation of renal hemodynamics, sodium excretion, and the aforementioned cardiovascular actions.

## Data Availability

The data presented in this study are available in https://docs.google.com/spreadsheets/d/1Iw3lQ6f1ZtMy7-XeEDoDXsyqbm6M-PGe/edit?usp=sharing&ouid=113469746811828789402&rtpof=true&sd=true (accessed on 22 March 2022).
